# Virtual phantom methodology for assessment of MRI distortion correction in high-precision stereotactic radiosurgery treatment planning

**DOI:** 10.3389/fonc.2025.1530332

**Published:** 2025-05-21

**Authors:** Tristan Belloeil-Marrane, Adrian Gutierrez, Marlies Boussaer, Cristina Teixeira, Thierry Gevaert, Mark De Ridder

**Affiliations:** Department of Radiotherapy, Universitair Ziekenhuis Brussel, Vrije Universiteit Brussel, Brussels, Belgium

**Keywords:** MRI distortion correction, stereotactic radiosurgery (SRS), cranial indications, target positioning accuracy, synthetic CT (sCT), magnetic resonance imaging (MRI), quality assurance (QA)

## Abstract

**Introduction:**

The accuracy of stereotactic treatment planning is primarily limited by the least accurate process in the whole chain of events, and is particularly important in cranial radiosurgery. Ameliorating this process can improve treatment targeting, providing additional reliability for these indications. Quality assurance (QA) in radiotherapy is often performed on the dose delivery and planning section rather than the localization. Magnetic Resonance Images (MRI) are notably subject to distortions, due to the nonlinearity of gradient fields, potentially source of geometric errors. This study aimed to analyze the impact of a patient-specific algorithm, rather than manufacturer-specific, to correct spatial distortion in cranial MRI by using a novel software-only paradigm.

**Material and methods:**

An unbiased simulated T1-Weighted MRI validated dataset is utilized to create a synthetic CT (sCT). By introducing controlled distortion in simulated datasets, we can evaluate the influence of noise and intensity non-uniformity (“RF”) ranging from 0 to 9% noise and 0 to 40% RF. These MRIs were corrected using the sCT as base modality for distortion correction. To evaluate the impact of the distortion correction, each corrected/non-corrected image set was compared to the unbiased MRI using Root-mean-square-error (RMSE) as a full-image reference comparison metric.

**Results:**

The distortion correction allows for an improvement based on the RMSE correlation between baseline and distorted MRIs. The amelioration of average RMSE in corrected versus non-corrected MRI is up to 42.22% for the most distorted datasets.

**Conclusion:**

The distortion correction results show a proportional improvement with increased noise and intensity non-uniformity. This provides additional robustness and reliability to the accuracy of SRS treatment planning using MR T1-W sequences as imaging reference for target definition and organ delineation, remaining consistent independently from the variability of the non-uniformity gradient values. This virtual phantom methodology primarily aims to provide a simple/robust evaluation metric in radiotherapy for MR distortion correction solutions, providing an additional/complement QA procedure to dedicated hardware phantoms, comparatively costly in time and resources. This approach is also designed to assist with an easily implementable secondary QA for validation during commissioning of distortion correction software, focusing on this feature, to better isolate and identify sources of geometric errors resulting from MR distortions.

## 1 Introduction

Magnetic Resonance Imaging (MRI) is one of the most utilized imaging modalities in Cranial Stereotactic Radiosurgery (SRS). Computed Tomography (CT) images are the gold standard in radiation oncology and are used for diagnostic, contouring, and dose calculation. This modality is considered to have superior spatial accuracy compared to MRI, but does not provide sufficient anatomical information for target definition and delineation of the organs at risk (OAR). MRI is required to enrich the treatment targeting in SRS treatments with sufficient anatomical data, thanks to an excellent soft tissue contrast (1).

During the treatment planning workflow, specifically within the contouring process, CT and MR images are rigidly fused to allow the projection of structures defined in one dataset to be displayed in the other. This registration is the key component to allow for simultaneous, accurate cranial structure contouring and dose calculation. MR images are prone to intrinsic distortion introduced during their acquisitions and might not be fully corrected by the MR scanner’s post-processing software. These remaining distortions can be a source of inaccuracy, resulting in potential incorrect target definition, sub-optimal protection of critical structures, and/or increased dose to normal tissue (2). The displacement of the treatment target linked to MRI distortions, mispositioning can lead to a geometric miss during delivery. This potential error is particularly crucial in SRS indications focusing on high-dose irradiation to small lesions, usually with a diameter inferior to 1cm. In these cases, a geometric deviation of 1mm or more could significantly impact the dose coverage of the target and, as well, increase the dose to the normal tissues. Moreover, the stereotactic target volume margins can be adjusted by increasing their size to compensate for potential geometric misses and guarantee sufficient dose coverage, leading to more than doubling the additional normal tissue volume receiving high doses for each 1mm margin increment. As a reference, for a sphere with a diameter of 1 cm, a 1mm margin will expand its initial volume by 33%, 73% with a 2mm margin, and 120% with a 3mm margin (3).

To verify accurate lesion targeting throughout SRS and SBRT treatments, the AAPM-RSS Medical Physics Practice Guideline 9.a (4). recommends an End-to-End (E2E) localization assessment “hidden target test” using an SRS frame and/or IGRT/SGRT system of 1 mm additionally stating that when developing the E2E tests, all aspects of the treatment process should be considered, including immobilization, simulation, respiratory management, treatment planning, and treatment delivery using a clinically relevant image guidance method. Systematic submillimeter E2E testing is necessary for SRS and requires continuous patient-specific quality assurance (QA), including discrete MRI correction distortion QA, considering the amplitude of potential displacement. For end-to-end testing, dedicated SRS-specific or anthropomorphic phantoms are typically used to define the overall error from image acquisition to radiation delivery However, the accuracy of a stereotactic treatment is primarily limited by the least accurate process in the whole chain of events. QA is often performed on the dose delivery and planning section rather than the target localization. The AAPM Task Group 284 (5) recommends a geometrical accuracy of ≤ 2 mm across a 25 cm field of view (FOV) for SRS and radiotherapy with MR-only planning.

During the SRS treatment process, the influence of the MRI inaccuracies associated with distortions persisting after initial scanner-level correction in the imaging QA is often overlooked and included in the broader treatment planning error. The principal challenge in asserting the accuracy of the MRI is that not all the distortions follow a linear gradient. Some distortions, the result of a gradient non-linearity, are referred to as B-spline distortions (6). Scanner manufacturers include some image reconstruction and correction processes during the post-processing of the images. Multiple software manufacturers have developed and validated elastic fusion in radiation oncology planning to improve the accuracy of target and critical organs definition. Institutions tend not to include distortion correction in their protocols, as the process remains a “black box” with little to no tools to assess the quality of the correction.

This study aims to define a new methodology based on a novel software-only paradigm. For this, we want to be software-agnostic and provide a robust and effortless technique that can be easily replicated in clinical institutions without requiring specific hardware and saving time and resources, particularly on medical imaging devices. This approach provided an adequate method to evaluate and prove the quality and effectiveness, as well as validate the clinical use of post-acquisition scanner processing distortion correction software using non-biased data along with appropriate metrics to comprehend the influence of the process on different defined distortion variables and how they are correlated.

## 2 Materials and methods

### 2.1 MR images and distortions generation

Since the geometrical displacement is non-linear, the incremental parameter should reflect it. For this purpose, BrainWeb MRI (7), an online interface that generates a set of realistic simulated brain databases (SBD), was utilized for a non-biased MRI simulation. This quantitative analysis of the image data approach was developed to provide a ground truth for such image sets to resolve the issue of validation for these sequences. The different parameters and values were estimated and validated to provide a realistic range of values to emulate the potential distortions generated during MR acquisition and provide a gradual set of determined variables to better quantify how distortions affect cerebral anatomy radiomics. The MR datasets anatomically encompass the integrality of the cranium, cerebrum, cerebellum, and brainstem from the top of the scalp to the base of the foramen magnum. To generate a high SNR ratio model, 27 high-resolution MR datasets of the same individual with normal anatomy were acquired, subsampled, and intensity averaged, resulting in a single simulated dataset (8–11).

MRI distortions consist of various hardware-related factors, such as magnetic field inhomogeneity and gradient non-linearity, along with patient-related aspects like chemical shift and magnetic susceptibility. Given the multiple variables, there is a consequent challenge in accurately identifying and attributing whether each one significantly impacts each specific image set and, moreover, in selecting optimal MR imaging parameters. To simulate a standard approach that does not depend on unique anatomical or environmental conditions, this study focuses on gradient non-linearity as the primary source of geometric distortions in this imaging modality (3). For that purpose, this study utilizes MRI datasets that were artificially generated using 2 variables: noise or percent noise (PN) (Gaussian noise percent multiplied by the brightest tissue intensity) and intensity non-uniformity (INU or radiofrequency (RF)) were introduced with defined incremental values in MR images to simulate the effects of distortions. The advantage of that technique is that the MR T1-Weighted (T1WI) dataset (PN=0%, RF=0%) used as the reference for correction distortion is considered “ground truth” (or gold standard) for the modality and is simulated from normal brain anatomy without distortion.

The INU fields were estimated from patient MRI scans. They are not linear but are slowly varying fields of a complex shape. The % value specifies the intensity non-uniformity level. For a 20% level, the multiplicative INU field has a range of values of [0.90 - 1.10] over the brain area. For other INU levels, the field is linearly scaled accordingly (i.e. to a range of [0.80 - 1.20] for a 40% level). INU distortions can be spatially smooth, not fully reproducing heterogeneous biological interactions within large anatomical structures. As a consequence, they can be easily interpreted and/or interpolated, which does not represent the clinical reality in MR imaging. To emulate the complexity of the imaging in the human head, incremental inhomogeneity magnitudes can be introduced to prevent automatic registration or segmentation from simply anticipating the INU distortions. For that purpose, Gaussian noise is specifically used to provide an additional layer of convolution to the INU (12). The noise parameter utilizes Rayleigh statistics in the background and Rician statistics in the signal regions. The PN number represents the percent ratio of the standard deviation of the white Gaussian noise versus the signal for a reference tissue. For the MR T1WI, the reference tissue for the noise computation used was the white matter.

For this study, all noise and RF values available in the BrainWeb model were analyzed: 17 simulated brain anatomy MRI T1WI datasets with 1x1x1 mm resolution and 181 slices using values ranging from 0 to 9% noise and from 0 to 40% RF.

### 2.2 Synthetic CT Conversion

In clinical routine, CT images are fused to the MRI for target and tissue density accuracy. However, they are subject to their intrinsic distortions and motion/reconstruction artifacts. The use of a synthetic CT (sCT) based on the validated non-distorted MRI was introduced to ensure that the CT images used as a base for correction distortion were not introducing any additional error in the process, particularly since using newly acquired CT from physical phantoms could still lead to some minor image processing geometrical errors during the acquisition and CT images would not match exactly with the MR images resulting in minor differences during the rigid registration. The sCT dataset was generated from the MRI baseline dataset (PN=0%, RF=0%) using a 1:1 voxel equivalence (13). The MRI baseline was segmented semi-automatically using ITK-SNAP 3.6.0 (14, 15) to differentiate grey and white matter, CSF, eyes, bone, air, and the rest of the soft tissues (16). The MR Arbitrary Units in each segment were then converted to Hounsfield Units (17) using bulk assignments and directly or inverse linear ranges as described in **Table 1**. To cope with the segmentation irregularities, a series of filters was applied, including a median filter, to ensure that the root fusion CT would mirror exactly the initial MR without distortion.

**Table 1:** Conversion table and methods from MR arbitrary units to Hounsfield Units for all defined tissue types used to generate a synthetic CT.

Tissue Type	MR Abitratry Units/HU Values Conversion Method	Hounsfield units (HU)
Bone	Inverse Linear	500 to 1100
Brain	Linear	35 to 75
Water (CSF, eyes)	Bulk Assignement	0
Soft tissues	Inverse Linear	−80 to −30
Air	Bulk Assignement	−1000

These values were slightly adapted from Yu et al. (15) and are in agreement with observed and published tissue density ranges.

### 2.3 MRI distortion correction

As part of the SRS treatment-specific planning solution currently utilized clinically in our institution, Elements Cranial Distortion Correction^©^ software version 4.0 (Brainlab AG, München, Germany) was selected and employed to correct the MR distortions remaining post-acquisition.

**Figure 1:**
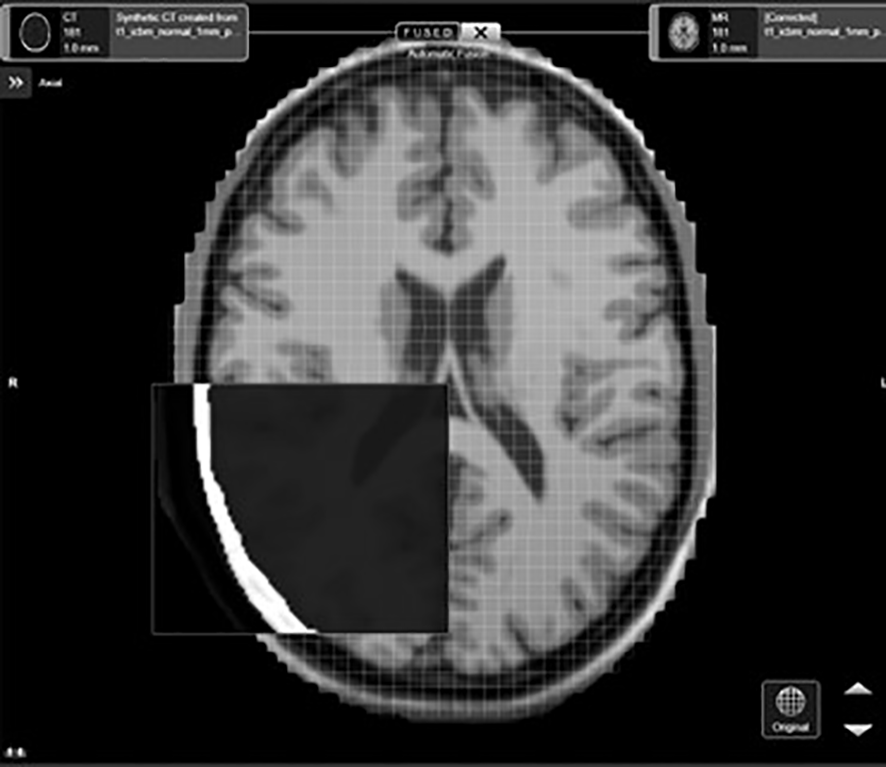
Display of the post-processed results of an MRI distorted dataset based on the synthetic CT using the cranial distortion correction software.

The sCT and simulated MRI datasets were imported in DICOM format and losslessly converted into a proprietary format. Within the software, the sCT is fused rigidly independently to all the MR datasets using mutual information registration. This process aligns the overall position of each MRI dataset to the CT positioning (no specific region of interest defined). The results of the fusion were verified visually using anatomical landmarks (e.g. ventricles, hemispherical midline, sulci,…) (**Figure 1**).

Following the initial rigid fusion, multiple rigid unit registrations of 3×3×3 cm3 overlapping units were performed within the images to locally improve the fusion. These units are then aggregated into a single deformation field that maps one of the datasets onto the other (18). This type of elastic registration can be referred to as “Multi-rigid” since multiple rigid fusions are applied at once, providing a blending of local registration results.

**Figure 2:**
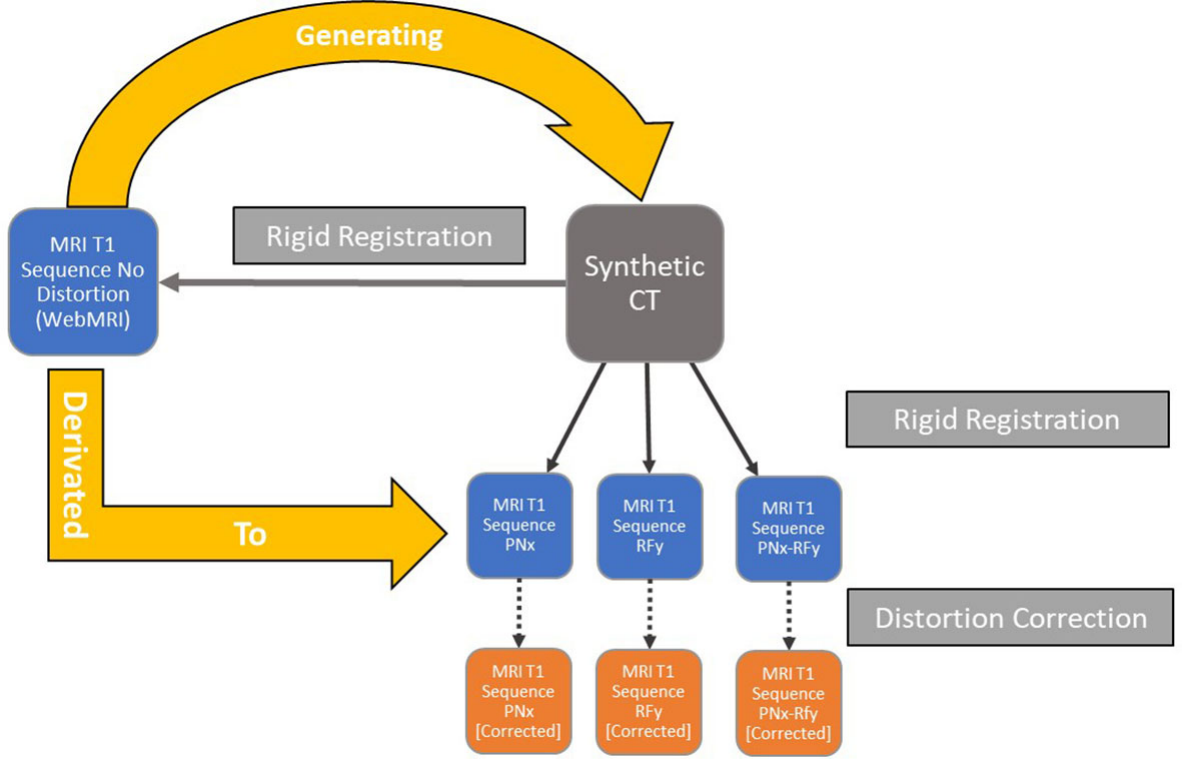
Workflow of the datasets’ generations and semi-rigid/rigid registrations for each iteration of the simulated data with different noise and intensity non-uniform parameters.

The multi-rigid fusions were applied independently for each MR Dataset in the distortion correction software using the sCT as a base for the distortion correction (**Figure 2**). The results of the registration were verified visually once more. The elastic registration software generates a corrected MRI dataset that can be exported while retaining the original MRI data. All the MR image datasets were exported in DICOM RT 3.0 format.

### 2.4 Evaluation of the correction

**Figure 3:**
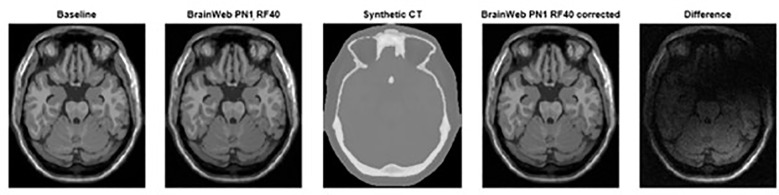
From left to Right, baseline MRI (PN0%, RF0%), distorted MRI (PN1%, RF40%), synthetic CT, and corrected distorted MRI (PN1%, RF40% - based on sCT correction) datasets including the difference observed using image subtraction between corrected and non-corrected datasets.

The root mean square error (RMSE) calculation is used to compare all the voxels (19) in the baseline MR against the corrected MR and the non-corrected MR for defined PN and RF values in the corresponding slice (**Figure 3**). In this study, the average RMSE was calculated using custom code in the MATLAB® software (MathWorks©, Natick, Massachusetts) to compare all the slices of the corrected to the non-corrected MR dataset according to the following equation:


$$RMSE=\;\sqrt{\sum_{i=1}^{n}\frac{{\left({\hat{y}}_{i}-y_{i}\right)}^{2}}{n}}$$

where $y_{i}$ is the value of a specific voxel in a selected MR image, ${\hat{y}}_{i}$ is the value of the aligned voxel in the baseline undistorted MR image and n is the number of voxels compared in the image.

### 2.5 Statistical analysis

A student t-test was performed to test differences between two groups/variables (corrected/non-corrected). Statistical significance was considered for p < 0.05.

## 3 Results

**Figure 4:**
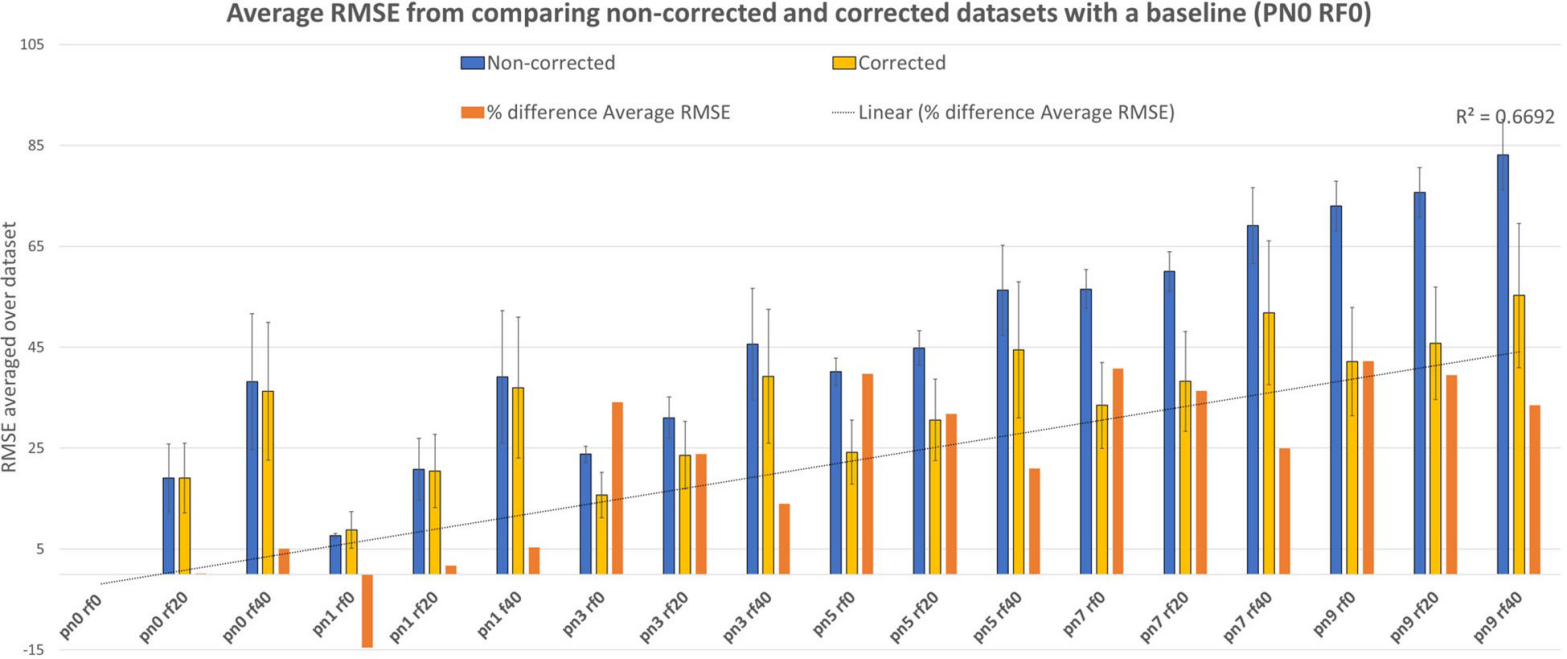
Average RMSE with linear trendline (R-squared = 0.67) comparing non-corrected and corrected datasets for PN% ranging from 0 to 9% and RF values ranging from 0 to 40% using a non-corrected baseline MRI (PN=0%, RF=0%).

On average, the distortion correction software allows for an improvement based on the RMSE correlation between the baseline MRI (PN=0%, RF=0%) and the distorted MRI corrected and non-corrected (**Figure 4**). For the corrected datasets, the RMSE ranges between 8.83 ± 0.27 and 55.58 ± 1.06 and averages at 33.36 ± 3.51. For the non-corrected datasets, the RMSE ranges between 7.70 ± 0.03 and 83.14 ± 0.52 and averages at 44.18 ± 5.61. A strong, statistically significant difference was found for the Average RMSE Corrected compared to Non-corrected, as observed in **Table 2**.

**Table 2:** Values of the average RMSE in 1 and percentage difference comparing non-corrected and corrected datasets for PN% ranging from 0 to 9% and RF values ranging from 0 to 40% using a non-corrected baseline MRI (PN=0%, RF=0%).

MRI Dataset	pn0 rf0	pn0 rf20	pn0 rf40	pn1 rf0	pn1 rf20	pn1 rf40	pn3 rf0	pn3 rf20	pn3 rf40	pn5 rf0	pn5 rf20	pn5 rf40	pn7 rf0	pn7 rf20	pn7 rf40	pn9 rf0	pn9 rf20	pn9 rf40
RMSE between non-corrected datasets																		
**Average Value**	**0**	**19.11**	**38.21**	**7.7**	**20.78**	**39.09**	**23.81**	**31.02**	**45.63**	**40.14**	**44.87**	**56.31**	**56.53**	**60.02**	**69.12**	**72.96**	**75.69**	**83.14**
**Standard Deviation**	**0**	**6.74**	**13.48**	**0.44**	**6.14**	**13.13**	**1.58**	**4.1**	**11.05**	**2.72**	**3.39**	**8.95**	**3.81**	**3.93**	**7.54**	**4.94**	**4.96**	**6.97**
**Minimum Value**	**0**	**6.89**	**13.75**	**7.05**	**10.89**	**16.13**	**21.74**	**24.58**	**29.65**	**37.05**	**38.98**	**42.3**	**52.41**	**54.01**	**56.3**	**67.98**	**69.02**	**70.95**
**Maximum Value**	**0**	**39.97**	**79.93**	**8.5**	**40.57**	**80.24**	**26.9**	**45.66**	**82.65**	**45.45**	**54.26**	**87.37**	**64.46**	**67.07**	**93.74**	**82.87**	**84.9**	**102.53**
RMSE between corrected datasets																		
**Average Value**	**0**	**19.07**	**36.28**	**8.83**	**20.43**	**37.01**	**15.69**	**23.61**	**39.25**	**24.19**	**30.61**	**44.51**	**33.49**	**38.22**	**51.86**	**42.16**	**45.81**	**55.25**
**Standard Deviation**	**0**	**6.92**	**13.68**	**3.62**	**7.27**	**13.94**	**4.49**	**6.66**	**13.28**	**6.34**	**8.07**	**13.48**	**8.52**	**9.91**	**14.26**	**10.72**	**11.15**	**14.3**
**Minimum Value**	**0**	**0**	**0**	**0**	**0**	**0**	**0**	**0**	**0**	**0**	**0**	**0**	**0**	**0**	**0**	**0**	**0**	**0**
**Maximum Value**	**0**	**34.65**	**66.85**	**22.77**	**38.58**	**67.23**	**27.36**	**37.19**	**67.95**	**35.61**	**44.57**	**69.97**	**40.97**	**49.6**	**75.02**	**53.32**	**57.67**	**75.37**
% difference RMSE between non-corrected and corrected																		
**Average Value**	**0**	**0.17**	**5.04**	**-14.56**	**1.68**	**5.32**	**34.11**	**23.87**	**13.98**	**39.74**	**31.79**	**20.95**	**40.75**	**36.32**	**24.97**	**42.22**	**39.48**	**33.55**
**Minimum Value**	**0**	**100**	**100**	**100**	**100**	**100**	**100**	**100**	**100**	**100**	**100**	**100**	**100**	**100**	**100**	**100**	**100**	**100**
**Maximum Value**	**0**	**13.31**	**16.37**	**-167.77**	**4.91**	**16.21**	**-1.72**	**18.53**	**17.79**	**21.65**	**17.86**	**19.92**	**36.44**	**26.04**	**19.97**	**35.66**	**32.07**	**26.49**

As a control, we measured the RMSE between the MRI baseline dataset (PN=0%, RF=0%) and its associated corrected MRI dataset. The average RMSE value was 14.01 (standard deviation SD 6.27) and serves as the baseline RMSE value comparing the undistorted reference images to other MR image sets.

The % difference of average RMSE in non-corrected versus corrected MRI ranges from 0 to 42.22%. A negative outlier (-14.56%) for the comparison at PN1, RF0 was found which was inconsistent with the rest of the data. This can be linked to the influence of the baseline value for lower values of PN and RF. The overall average, including the outlier value, was 21.08 ± 4.16%. This confirms the overall increased accuracy, and the linear trendline (**Table 2**) shows a positive correlation with PN and RF values (R2 = 0.67).

### 3.1 RF correction

The combined averaged PN values from 0 to 9% for each RF value were calculated to evaluate the influence of distortion correction on the RF% %. For the corrected datasets, the RMSE ranges between 20.73 (SD 15.69) and 44.02 (SD 7.99) and averages at 31.46 ± 6.79 (SD 11.45). For the non-corrected datasets, the RMSE ranges between 33.52 (SD 28.33) and 55.25 (SD 17.98) and averages at 43.56 ± 6.33 (SD 22.98). The results between non-corrected and corrected RMSE average percentages showed a significant improvement for all RF values: RF0% = 38.18%, RF20% = 31.72%, and RF40% = 22.30% with an average of 29.27% ± 5.16%. Statistical significance (p=0.014) was found for the compared RF RMSE values.

### 3.2 PN correction

The combined averaged RF=0% RMSE for each PN value was calculated to evaluate the influence of distortion correction on the PN% %. For the corrected datasets, the RMSE ranges between 8.83 and 55.58, and averages at 20.73 ± 6.41 (SD 15.69). For the non-corrected datasets, the RMSE ranges between 7.70 and 72.96, and averages at 33.52 ± 11.57 (SD 28.33). The results between non-corrected and corrected RMSE average percentages showed significant improvements (p= 0.026) for PN values equal and superior to 1%: PN0 = 0% PN1 = -14.56%, PN3 = 34.11%, PN5 = 39.74%, PN7 = 40.75% and, PN9 = 42.22% with an average of 23.71 ± 10.04%.

## 4 Discussion

The premises of our study were formulated on the lack of a consistent and easy-to-implement clinical method to assess the quality of the distortion correction in MRI. The currently available options require having a dedicated MR phantom and acquiring the image data on CT and MRI scanners (20). This methodology was implemented to address this specific issue and independently assess the specific process of MRI distortion correction.

Accurate target definition is always crucial for cranial SRS as it will influence the overall treatment accuracy since the targeted volumes are small, typically ranging from 0.3cc to 50cc (21, 22), and are heavily dependent on MRI. This is even more predominant for functional SRS: Luo et al. (23) compared the positioning of the SRS treatment isocenter with the ventral intermediate (VIM) nucleus of the thalamus during thalamotomies and the tremor treatment response post-irradiation, concluding on the critical importance of submillimetric accuracy for these specific indications.

That is one of the reasons assessing the effect of the distortion correction is primordial to avoid inaccurate treatment delivery leading to diminished response and normal tissue toxicity.

Our results have shown that the Elements Cranial Distortion Correction^©^ software was improving the accuracy of target and critical structures delineation in MR T1 sequences by mitigating geometric misses resulting from gradient nonlinearity distortions. According to the full-image RMSE results, the distortion correction is positively correlated to the simultaneous increment of the PN and RF. Moreover, the more the datasets are distorted, the more efficient the software will be. These correction effects were also observable for respectively augmenting the PN (average: 23.71% for Non-Corrected vs. Corrected datasets) and RF values (average: 29.27% for Non-Corrected vs. Corrected datasets) in the MR images. This supports an added reliability to the quality of the correction independently from the type of distortion in MR T1WI images.

Our results align with the results of the clinical validation of the software by the manufacturer where the spatial correlation between rigidly and elastically fused images was assessed through Euclidean distance, They found an improvement in fusion accuracy of the 1.5/3.0T MRI by 0.41 ± 0.95 mm (18).

The results of our study showed non-significant improvements for lower PN and RF % values correlated to the notable baseline observed using the RMSE value (14.01) from the MRI baseline datasets (PN=0%, RF=0%), inferior to the average standard deviation for both corrected and non-corrected datasets with their respective RMSE values of 14.06 and 25.93. This is most likely related to the limit of the distortion correction accuracy. Karger et al. (2006) (24) reported the radial distance correction with device-specific 2D and 3D image distortion correction algorithms in multiple scanners with different magnetic B-field strengths in Tesla (T) for cranial indications. They concluded that the image distortions superior to 2 mm were significantly reduced, but not significantly for distortions inferior to 2 mm due to gradient non-linearities. Bagherimofidi et al. (2019) (25) have reported similar findings using their specific distortion correction algorithm in a head phantom: the average error varied from 0.258 to 0.557 mm with a maximum error of 1.492 mm with diameter distances from 20 to 80 mm from the isocenter, confirming a baseline error post- correction distortion. The study of Retif et al. also aligns with our results as it was demonstrated in phantom and clinical patient data that the Elements Distortion Correction software was able to reduce the mean and standard deviation datasets, particularly in the maximum distortion in heavily distorted images, significantly reducing the number of control points with > 0.5-mm distortion. Furthermore, these results were consistent across acquisitions from different scanner makes, models, and magnetic field strengths (26). Image quality metrics (IQMs) such as RMSE and structural similarity index (SSIM) were the core processes for assessing the correction direction using a full-reference quality metric (27). These are commonly used in the evaluation and optimization of MRI acquisition and reconstruction strategies, including MRI distortion measurements. Root Mean Square Error (RMSE) is utilized in diverse fields of study to compare 2 images and has been a standard metric in medical images (28). The advantages and justifications of this measurement tool in the scope of our study were its ease of use, rapid calculation time, and robustness of results, permitting for more simple and accessible testing. More importantly, it allows us to compare the quality of the entire image to a baseline and, therefore is more inclusive than other methods. This is particularly important as the distortions are non-linear and comparison of points or structures could be misleading since they do not provide an overview of the image and might lead to misinterpretation of the quality of the dataset.

This is also a better fit for the verification of the MRI distortion correction as distortions are not uniform and punctual evaluation can lead to misinterpretation of the absolute local values and/or large amplitude between the minimum and maximum deviations. In the absence of fiducials or implanted markers, comparing anatomical landmarks or delineations of specific organs, due to the non-uniformity nature of the MR distortions, these values can be significantly different depending on the anatomical position of the sampled landmark or contour and do not reflect the influence of distortions for the entire image set or even in other anatomical cranial regions. Moreover, the operator can introduce additional errors when comparing or delineating organs, which can be further accentuated when multiple operators are involved. Intra- and inter-operator variabilities are common issues in assessing the quality of organ contouring and can be avoided by using a full-image index of similarity such as the RMSE.

Regarding the choice of metrics, out of all the IQMs, the RMSE was the most efficient and simple to implement. The other commonly used IQM: SSIM is also a strong measuring metric, that, however, requires a more complex implementation and calculating power. Based on the study of Mason et al. (28), the simple and rapid algorithms of RMSE demonstrated short calculation times (all less than 2 seconds). SSIM has slightly longer calculation times (less than 20 seconds). It also demonstrated that SSIM does not show a significantly stronger correlation with radiologists’ observation of diagnostic image quality than RMSE.

This assessment method is not limited to the virtual phantom and can be applied similarly to hardware phantoms. These highly specialized apparatuses may already be accepted as the standard for process validation and periodic verification in the established QA protocol; the technique described in this study would grant an additional level of reliability and a secondary check to supplement the existing approach. This would further ensure the accuracy and consistency of the measurement. In addition, both virtual and hardware can be used concurrently following this method to the same purpose, adding the benefit of a strong correlation as a result of relying on a common metric to establish the validity and effectiveness of the distortion correction.

In the radiation oncology treatment planning, the CT and MRI T1WI are the 2 imaging modalities mandatorily included for all cranial indications. This study focused on T1-weighted imaging for this reason. For specific SRS cranial indications, other sequences are required, such as T2-weighted images (29). We aim to evaluate the quality of the distortion correction in subsequent studies using this measurement method and validate the software for all possible cranial MRI sequences.

Papas et al. (2017) (30) quantified the influence of distortion correction in RT planning by employing a phantom study. They concluded that for targets inferior to 20 mm in diameter, spatial disposition of the order of 1 mm could significantly affect plan acceptance/quality indices. For targets with a diameter greater than 2cm, the corresponding disposition was found to be greater than 1.5mm. It underlines the relevance of target accuracy in SRS treatment delivery. This effect is magnified in treatments of simultaneous multiple lesions with a single isocenter; translational and rotational deviations of isocenter as small as 0.5 mm and 0.5 deg. in the treatment delivery could lead to significant dosimetric impact as suggested by literature (31–33). For such indications, the distortion correction process would help to improve the accuracy and, logically, decrease the margin needed to treat the metastasis to preserve normal brain tissue.

This current study has reviewed a specific distortion algorithm based on multi-rigid registration. Other commercial software is available and makes use of different methods of elastic fusion. The continuation goal of our study is to use the newly defined method to further evaluate other distortion correction algorithms or techniques and provide a comprehensive comparison with different software and modalities for cranial SRS and MR-only treatment planning. We aim that these kinds of distortion correction algorithms will become more and more important with the emergence of 7T scanners, providing higher signal-to-noise ratio, spatial resolution, and contrast for clinical applications in SRS and neurosurgery (34). It has been shown in the literature that increasing the magnetic B-field strength (T) was correlated to increased distortions (35).

The ease of use and availability of the data for the implementation of this QA method can be further utilized in parallel to other available QA options, including those relying on an MR-dedicated phantom, offering the possibility of a secondary validation and providing additional details on the distortion correction process. Future studies will aim to assess the correlation between the different approaches and further investigate the complementarity of different QA techniques to improve the commissioning, validation, and daily verification of distortion correction processes in SRS treatment planning as well as introduce other MRI acquisition variables to assess their influence in the quality of the distortion correction and ensure that all QA methods align on results.

## 5 Conclusion

The distortion correction of MR T1-weighted images is a requirement to add robustness and reliability to the target definition ensuring accurate and consistent cranial treatment planning, particularly for SRS indications. The described distortion correction evaluation method based on non-biased datasets with defined parameter values and relying on standard medical image quality metrics has demonstrated the facility of isolating and assessing the quality of this specialized process with simple software tools available for every institution. With this novel approach using a simulated virtual phantom, we are able to provide additional validation related to the image datasets’ accuracy needed for dedicated cranial indications in radiosurgery, MR-only treatments as well as neurosurgical functional indications, and can be further utilized alongside other QA methods to add a secondary validation/verification method.

## Data Availability

The original contributions presented in the study are included in the article/supplementary material. Further inquiries can be directed to the corresponding author/s.
